# (−)-Epigallocatechin-3-Gallate Enhances Hepatitis C Virus Double-Stranded RNA Intermediates-Triggered Innate Immune Responses in Hepatocytes

**DOI:** 10.1038/srep21595

**Published:** 2016-02-16

**Authors:** Yizhong Wang, Jieliang Li, Xu Wang, Juliet C. Peña, Kui Li, Ting Zhang, Wenzhe Ho

**Affiliations:** 1 Department of Infectious Diseases, Shanghai Children’s Hospital, Shanghai Jiao Tong University, Shanghai 200040, P.R. China; 2 Department of Pathology and Laboratory Medicine, Temple University School of Medicine, Philadelphia, PA 19140, USA; 3 Deparment of Microbiology, Immunology, and Biochemistry, University of Tennessee Health Science Center, Memphis, TN 38163, USA

## Abstract

(−)-Epigallocatechin-3-gallate (EGCG), a major polyphenol component of green tea, has recently been identified as an inhibitor of hepatitis C virus (HCV) entry. Here, we examined whether EGCG can enhance hepatocyte-mediated intracellular innate immunity against HCV. HCV dsRNAs (Core, E1-P7, NS-3′NTR and NS5A) induced interferon-λ1 (IFN-λ1) expression in human hepatocytes. These HCV dsRNAs also induced the expression of Toll-like receptor 3 (TLR3), retinoic acid-inducible gene I (RIG-I) and several antiviral IFN-stimulated genes (ISGs) expression. Although EGCG treatment of hepatocytes alone had little effect on TLR3 and RIG-I signaling pathways, EGCG significantly enhanced HCV dsRNAs-induced the expression of IFN-λ1, TLR3, RIG-I and antiviral ISGs in hepatocytes. Furthermore, treatment of HCV-infected hepatocytes with EGCG and HCV dsRNAs inhibited viral replication. Given that EGCG has the ability to enhance HCV dsRNAs-induced intracellular antiviral innate immunity against HCV, suggesting the potential application of EGCG as a new anti-HCV agent for HCV therapy.

Hepatitis C virus (HCV) infection is a major cause of chronic hepatitis, liver cirrhosis and hepatocellular carcinoma^1^. The interactions between HCV and the host immune system in the liver play a key role in the immunopathogenesis of HCV disease. The clinical outcome of HCV infection and the degree of liver damage are the results of complicated interactions between the virus and host immune responses^2^. Although cellular and humoral immune responses are present during acute and chronic HCV infection, these immune responses appear to be ineffective in eradicating the virus^3^. Studies of HCV-host interactions have revealed that HCV can use several approaches to compromise the host immune response during viral infection^4^. For example, HCV NS3/4A protease can impair Toll-like receptor 3 (TLR3) and retinoic acid-inducible gene I (RIG-I) signaling, resulting in the inhibition of interferon (IFN) regulatory factor 3 (IRF-3) activation and IFN-β expression^5^,^6^,^7^.

Green tea has been considered to have a number of physiological and pharmacological health benefits. (−)-Epigallocatechin-3-gallate (EGCG) is the most abundant and bioactive catechin in green tea, which displays strong preventive effects against viral infection, cardiovascular disease, metabolic syndrome, neurodegenerative diseases and cancer^8^,^9^. EGCG has antiviral activities on diverse families of viruses, including HIV^9^, human herpes simplex virus (HSV)^10^, and influenza virus^11^. As a potent antioxidant, EGCG has been shown to have both anti-inflammatory and anti-atherogenic properties^12^,^13^,^14^. We previously^14^ showed that EGCG inhibits endotoxin-induced expression of inflammatory cytokines in human cerebral microvascular endothelial cells. Studies^15^,^16^ showed that EGCG has strong anticancer properties through inhibiting proteasome activity and inducing ER stress. It was also reported that EGCG increases lipid-droplet formation and impairs very-low-density lipoprotein secretion in hepatocytes^17^. Studies^18^,^19^ also showed that EGCG can act as an inhibitor of HCV entry. While EGCG dose not interfere with HCV genome replication and egress, EGCG blocks an early step of the HCV entry process and inhibits HCV cell-to-cell transmission^18^,^19^.

In this study, we examined whether EGCG possesses the ability to enhance intracellular innate immunity against HCV in hepatocytes. Our results revealed that EGCG significantly increases HCV dsRNAs-induced IFN-λ1, TLR3, RIG-I, and antiviral IFN-stimulated genes (ISGs) expression in both HCV JFH-1-infected and uninfected Huh7 cells. More importantly, the combination of EGCG treatment and the exposure of HCV dsRNAs to hepatocytes inhibits HCV replication.

## Results

### HCV dsRNAs induce IFN-λ1

**Figure 1:**
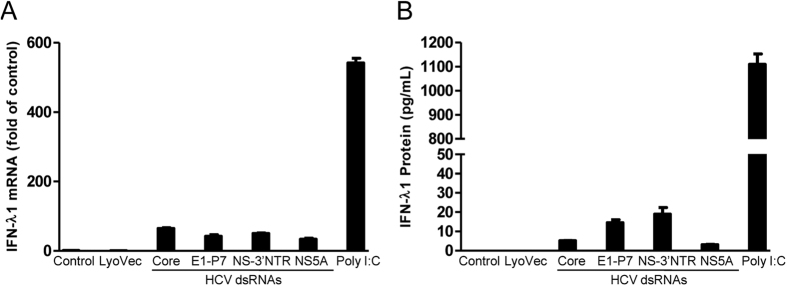
HCV dsRNAs induce IFN-λ1 expression. Huh7 cells were stimulated with the HCV dsRNAs (Core, E1-P7, NS-3′NTR, NS5A, 1 μg/mL), same dose of poly I:C (1 μg/mL) was used as a positive control. Total RNA extracted from cells after 24 h exposure was subjected to the real time RT-PCR for the mRNA levels of IFN-λ1 and GAPDH. The data are expressed IFN-λ1 mRNA (**A**) levels relative (fold) to the control (vehicle only, which defined as 1). Supernatant (SN) was collected from the cell cultures 48 h post exposure for ELISA to measure the protein levels of IFN-λ1 (**B**). The results shown are mean ± SD of triplicate, representative of three independent experiments.

**Figure 2:**
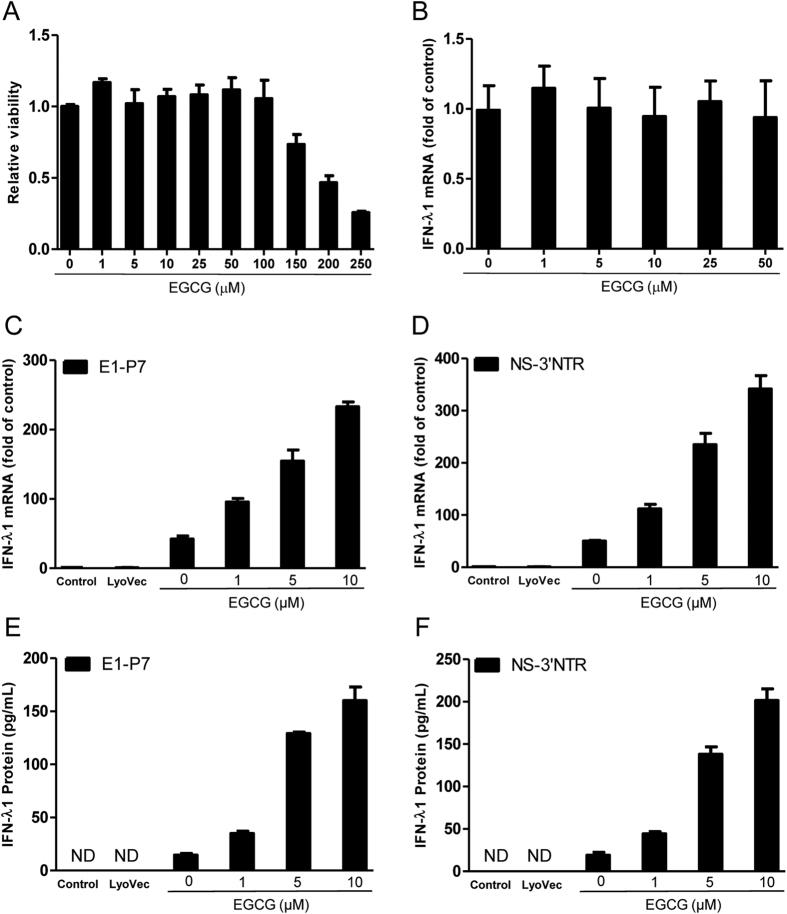
EGCG enhances HCV dsRNAs-induced IFN-λ1. (**A**) Cyotoxicity test of EGCG on Huh7 cells. Huh7 cells were treated with concentrations of EGCG as indicated for 72 h. The cells viability was determined using an MTS-based viability assay by determining OD at 490 nm. (**B**) Effect of EGCG on IFN-λ1 expression in Huh7 cells. Huh7 cells were treated with EGCG at the indicated concentrations for 24 h. Total RNA from cells was subjected to IFN-λ1 gene expression by real time RT-PCR. (**C–F**) Effect of EGCG on dsRNAs-induced IFN-λ1 expression in Huh7 cells. Huh7 cells were treated with EGCG as indicated for 1 h prior to HCV dsRNAs (E1-P7, NS-3′NTR) stimulation. Total RNA extracted from cells after 24 h stimulation was subjected to the real time RT-PCR for the mRNA levels of IFN-λ1 and GAPDH. The data are expressed IFN-λ1 mRNA (**C,D**) levels relative (fold) to the control (vehicle only, which defined as 1). After 48 h stimulation, supernatant (SN) was collected from the cell cultures for ELISA to measure the protein level of IFN-λ1 (**E,F**). The results shown are mean ± SD of triplicate, representative of three independent experiments.

To test the effect of HCV dsRNAs on IFN-λ1 expression in Huh7 cells, we stimulated Huh7 cells with the HCV dsRNAs (Core, E1-P7, NS-3′NTR and NS5A, 1 μg/mL). As shown in Fig. 1A, Core, E1-P7, NS-3′NTR and NS5A dsRNA induced IFN-λ1 mRNA expression. In addition, these HCV dsRNAs induced the IFN-λ1 expression at the protein level (Fig. 2A). Although we found that the HCV dsRNAs induced the expression of IFN-λ1 at both mRNA and protein levels, the induction level of IFN-λ1 was much lower than with the same dose (1 μg/mL) stimulation of poly I:C.

### EGCG enhances HCV dsRNAs-induced IFN-λ1

We first determined the cytotoxicity effect of EGCG on Huh7 cells. As shown in Fig. 2A, although cytotoxicity of EGCG was found at concentration of 150 μM after 72 h post treatment, the concentrations at 100 μM or lower had little cytotoxicity on Huh7 cells. We then examined the effect of EGCG on IFN-λ1 expression in Huh7 cells. We showed that EGCG treatment of Huh7 cells had little effect on IFN-λ1 expression in Huh7 cells (Fig. 2B). In contrast, EGCG pretreatment could significantly and dose-dependently enhance the HCV dsRNAs-induced IFN-λ1 expression in Huh7 cells at both mRNA (Fig. 2C,D, Fig. S1A,B) and protein (Fig. 2E,F, Fig. S1C) levels.

### EGCG enhances HCV dsRNAs-induced TLR3 and RIG-I

**Figure 3:**
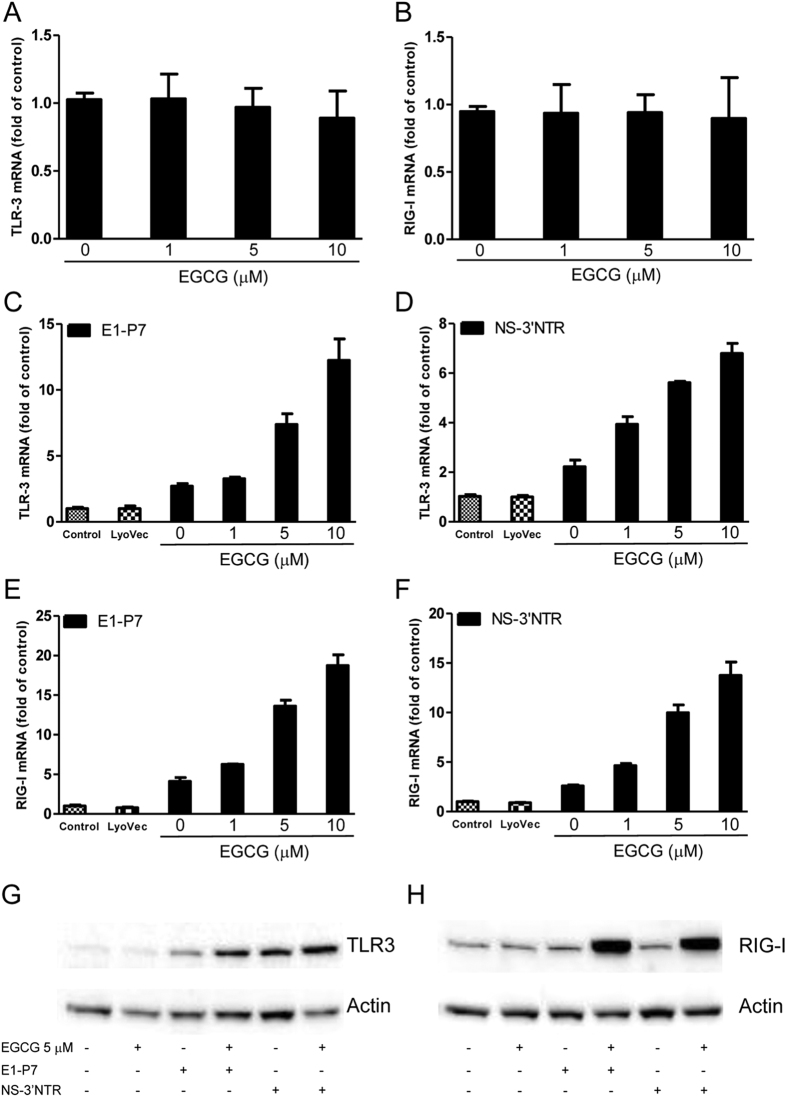
EGCG increases HCV dsRNAs-induced TLR3 and RIG-I expression. (**A,B**) Effect of EGCG on TLR3 and RIG-I expression in Huh7 cells. Huh7 cells were treated with EGCG at the indicated concentrations for 24 h. Total RNA extracted from cells was subjected to TLR3 (**A**) and RIG-I (**B**) gene expression by real time RT-PCR. (**C–G**) Effect of EGCG on dsRNAs-induced TLR3 and RIG-I expression in Huh7 cells. Huh7 cells were treated with EGCG as indicated for 1 h prior to HCV dsRNAs (E1-P7, NS-3′NTR) stimulation. Total RNA extracted from cells after 24 h stimulation was subjected to the real time RT-PCR for the mRNA levels of TLR3, RIG-I and GAPDH. The data are expressed as TLR3 (**C,D**) and RIG-I mRNA (**E,F**) levels relative (fold) to the control (vehicle only, which defined as 1). The results shown are mean ± SD of triplicate, representative of three independent experiments. After 48 h stimulation, cell lysates were collected from the cell cultures 48 h after stimulation for Western blot to measure the protein level of TLR3 (**G**) and RIG-I (**H**). Three independent experiments were performed and one representative experiment is shown.

Similarly, while EGCG alone had little effect on the expression of TLR3 (Fig. 3A) and RIG-I (Fig. 3B), EGCG pretreatment could significantly enhance HCV dsRNAs-induced TLR3 (Fig. 3C,D, Fig. S2A,B) and RIG-I (Fig. 3E, F, Fig. S2C,D) mRNA expression in Huh7 cells, which was confirmed by Western blot using specific antibodies against TLR3 (Fig. 3G, Fig. S2E) and RIG-I (Fig. 3H, Fig. S2F).

### EGCG enhances HCV dsRNAs-induced ISGs

**Figure 4:**
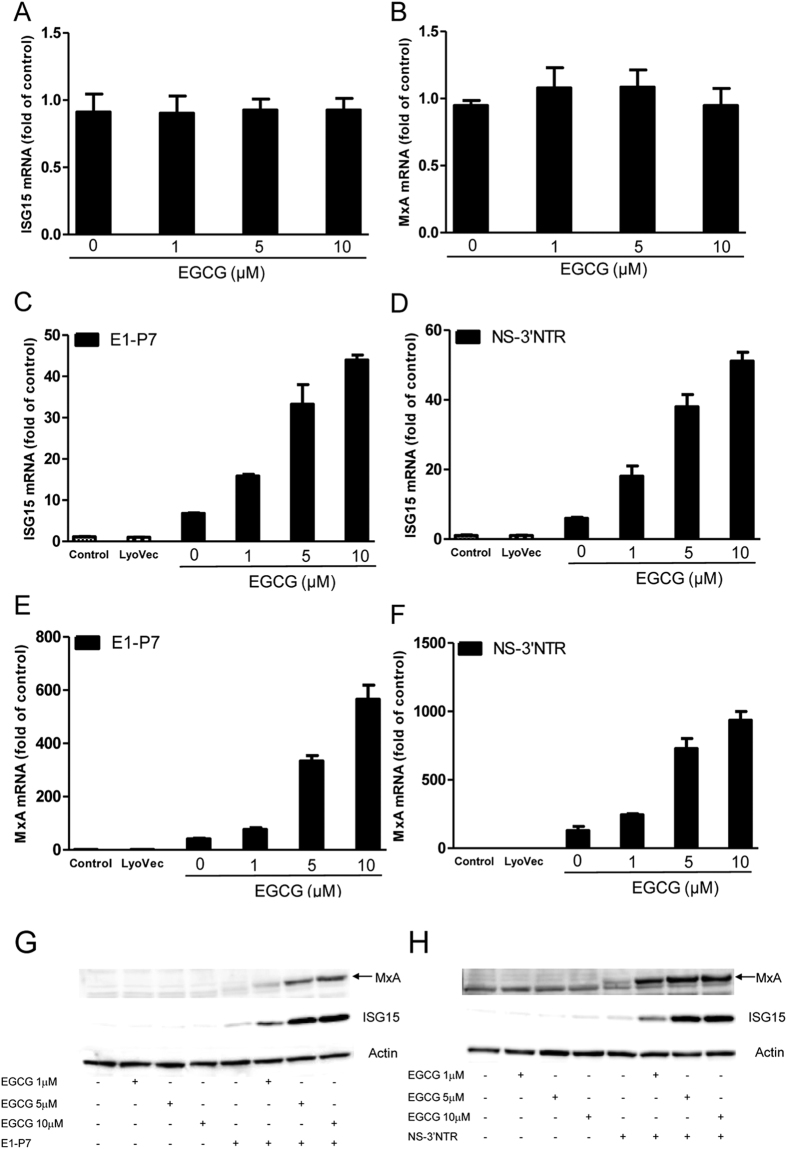
EGCG enhances HCV dsRNAs-induced ISGs expression in Huh7 cells. (**A,B**) Effect of EGCG on ISG15 and MxA expression in Huh7 cells. Huh7 cells were treated with EGCG at the indicated concentrations for 24 h. Total RNA from cells was subjected to ISG15 (**A**) and MxA (**B**) gene expression by real time RT-PCR. (**C–H**) Effect of EGCG on dsRNAs-induced ISG15 and MxA expression in Huh7 cells. Huh7 cells were treated with EGCG as indicated for 1 h prior to HCV dsRNAs (E1-P7, NS-3′NTR) stimulation. Total RNA extracted from cells 24 h after stimulation was subjected to the real time RT-PCR for the mRNA levels of ISG15 and MxA and GAPDH. The data are expressed ISG15 (**C,D**) and MxA mRNA (**E,F**) levels relative (fold) to the control (vehicle only, which defined as 1). The results shown are mean ± SD of triplicate, representative of three experiments. Cell lysates were collected from the cell cultures 48 h after stimulation for Western blot to measure the protein level of ISG15 and MxA (**G,H**). Three independent experiments were performed and one representative work is shown.

We determined whether EGCG treatment and/or HCV dsRNAs stimulation can induce ISGs expression in Huh7 cells. EGCG alone had little effect on the expression of ISG15 (Fig. 4A) and MxA (Fig. 4B). In contrast, HCV dsRNAs induced the expression of ISG15 (Fig. 4C,D, Fig. S3A,B) and MxA (Fig. 4E,F, Fig. S3C,D) in Huh7 cells at both mRNA (Fig. 3B,C,E,F, Fig. S3A,B) and protein (Fig. 4G,H, Fig. S3E) levels. We next examined the effect of EGCG on HCV dsRNAs-induced ISG15 and MxA expression in Huh7 cells. As shown in Fig. 3B–H and Fig. S3, EGCG dose-dependently enhanced the HCV dsRNAs-induced ISG 15 and MxA expression at both mRNA and protein levels.

### Treatment with both EGCG and HCV dsRNAs inhibits HCV

**Figure 5:**
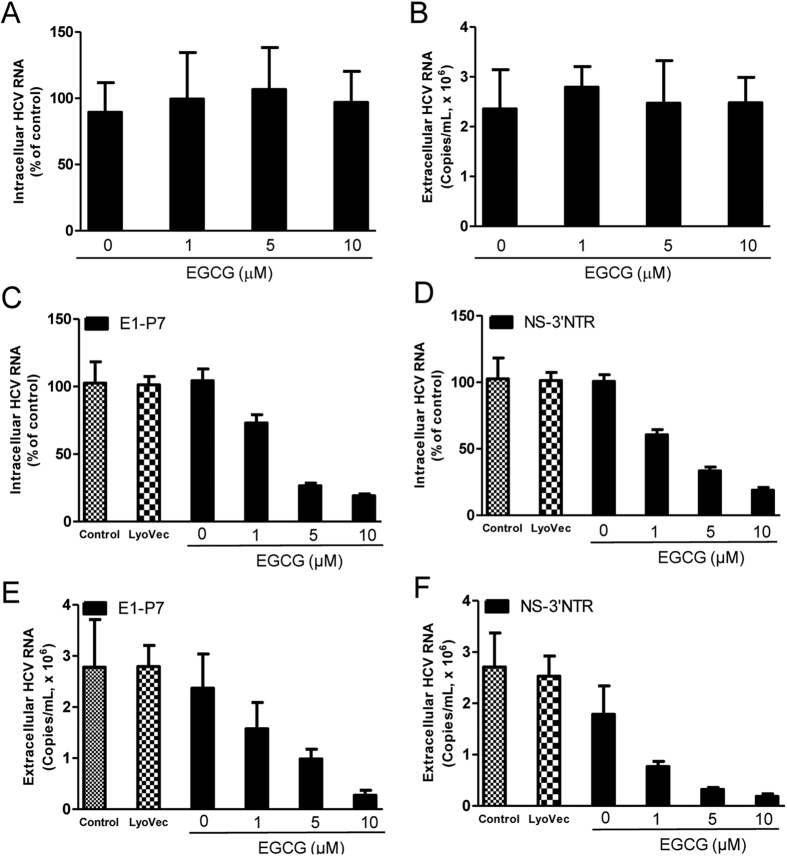
The combination of EGCG treatment and HCV dsRNAs stimulation inhibits HCV replication. (**A,B**) Effect of EGCG on HCV JFH-1 replication. JFH-1-infected Huh7 cells (72 h postinfection) were treated with EGCG at the indicated concentrations for 48 h. Intracellular (**A**) and extracellular (**B**) RNA extracted from JFH-1-infected Huh7 cells or culture supernatant (SN) was subjected to the real time RT-PCR for HCV and GAPDH RNA quantification. (**C–G**) Effect of the combination of EGCG treatment and HCV dsRNAs stimulation on HCV replication. JFH-1-infected Huh7 cells (72 h postinfection) were treated with EGCG at the indicated concentrations for 1 h prior to HCV dsRNAs (E1-P7, NS-3′NTR) stimulation. Intracellular (**C,D**) and extracellular (**E,F**) RNA was extracted from JFH-1-infected Huh7 cells or culture SN after 48 h stimulation was subjected to the real time RT-PCR for HCV and GAPDH RNA quantification. Intracellular HCV RNA level is expressed as HCV RNA levels relative (%) to the control (vehicle only, which are defined as 100%). Extracellular is expressed as copies/mL. The results shown are mean ± SD of triplicate cultures, representative of three experiments.

EGCG treatment alone had little effect on HCV JFH-1 replication at a concentration under 10 μM (Fig. 5A,B), and HCV dsRNAs (E1-P7, NS-3′NTR) treatment also had limited anti-HCV effect in Huh7 cells (Fig. 5C–F). When we treated JFH-1-infected cells with EGCG for 1 h prior to HCV dsRNAs stimulation, we observed that EGCG treatment combined with HCV dsRNAs exposure inhibited HCV replication in Huh7 cells, and the EGCG effect was dose-dependent (Fig. 5C–F). This HCV inhibition mediated by EGCG and HCV dsRNAs was confirmed both by intracellular (Fig. 5C,D) and extracellular (Fig. 5E,F) HCV RNA levels.

### EGCG enhances HCV dsRNAs-induced IFN-λ1 in HCV-infected cells

**Figure 6:**
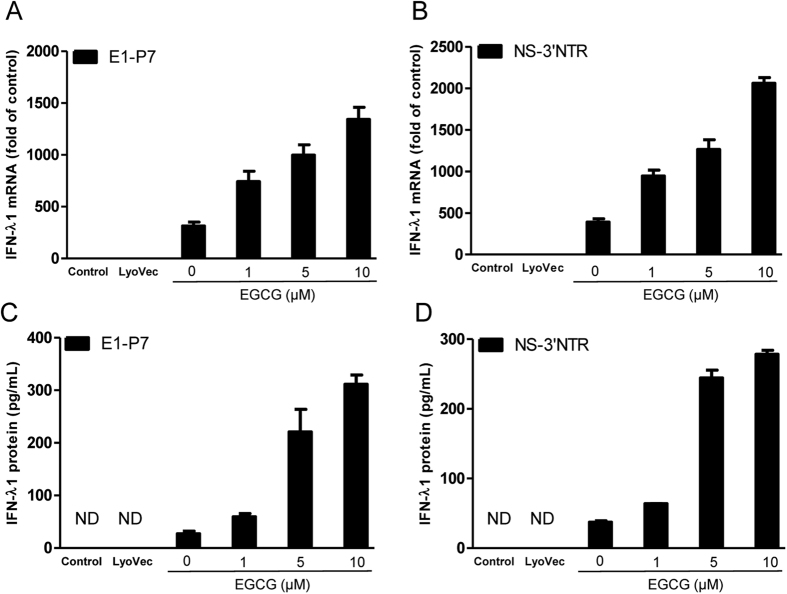
Effect of EGCG on HCV dsRNAs-induced IFN-λ1 expression in JFH-1-infected Huh7 cells. JFH-1-infected Huh7 cells (72 h postinfection) were treated with EGCG as indicated for 1 h prior to HCV dsRNAs (E1-P7, NS-3′NTR) stimulation. Total RNA extracted from cells after 24 h stimulation was subjected to the real time RT-PCR for the mRNA levels of IFN-λ1 and GAPDH. The data are expressed IFN-λ1 mRNA (**A,B**) levels relative (fold) to the control (vehicle only, which defined as 1). After 48 h stimulation, supernatant (SN) was collected from the cell cultures for ELISA to measure the protein level of IFN-λ1 (**C,D**). The results shown are mean ± SD of triplicate, representative of three experiments.

To investigate the mechanisms of EGCG and HCV dsRNAs-mediated HCV inhibition, we examined the effect of EGCG on HCV dsRNAs-induced IFN-λ1 expression in JFH-1-infected Huh7 cells. We found that EGCG treatment also had little effect on IFN-λ1 expression in JFH-1-infected Huh7 cells (data not show). HCV dsRNAs stimulation alone could induce IFN-λ1 expression at both mRNA (Fig. 6A,B) and protein (Fig. 6C,D) levels in JFH-1-infected cells. When we treated cells with EGCG for 1 h prior to HCV dsRNAs stimulation, we showed that the EGCG treatment can significantly enhance the HCV dsRNAs-induced IFN-λ1 expression in JFH-1-infected Huh7 cells, at both mRNA (Fig. 6A,B) and protein levels (Fig. 6C,D), and this effect was dose-dependent.

### EGCG enhances HCV dsRNAs-induced ISGs in HCV-infected cells

**Figure 7:**
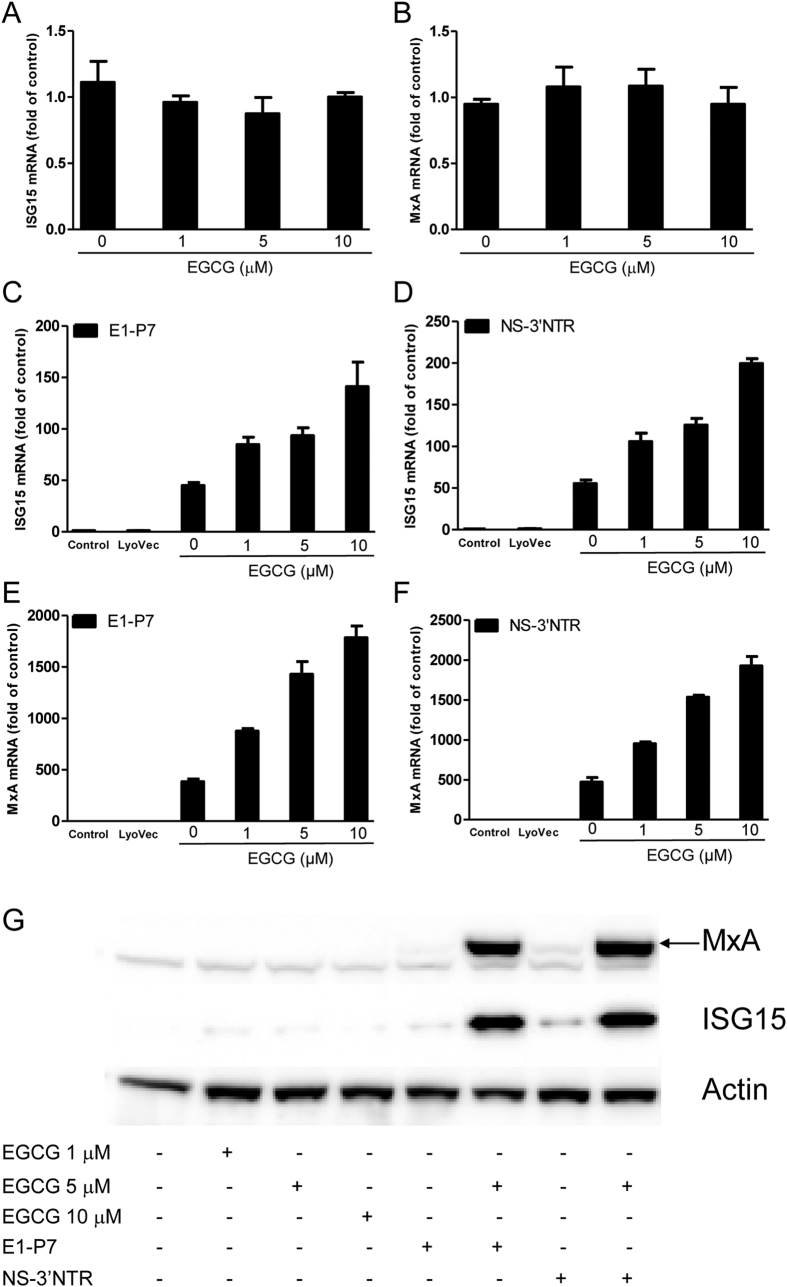
Effect of EGCG on HCV dsRNAs-induced ISGs expression in JFH-1-infected Huh7 cells. (**A,B**) Effect of EGCG on ISG15 and MxA expression in JFH-1- infected Huh7 cells. JFH-1-infected Huh7 cells (72 h postinfection) were treated with EGCG at the indicated concentrations for 24 h. Total RNA from cells was subjected to ISG15 (**A**) and MxA (**B**) gene expression by real time RT-PCR. (**C–H**) EGCG enhances dsRNAs-induced ISG15 and MxA expression in JFH-1-infected Huh7 cells. JFH-1-infected Huh7 cells (72 h postinfection) were treated with EGCG as indicated for 1 h prior to HCV dsRNAs (E1-P7, NS-3′NTR) stimulation. Total RNA extracted from cells after 24 h stimulation was subjected to the real time RT-PCR for the mRNA levels of ISG15 and MxA and GAPDH. The data are expressed ISG15 (**C,D**) and MxA mRNA (**E,F**) levels relative (fold) to the control (vehicle only, which defined as 1). The results shown are mean ± SD of triplicate, representative of three experiments. After 48 h stimulation, cell lysates were collected from the cell cultures for Western blot to measure the protein level of ISG15 and MxA (**G**). Three independent experiments were performed and one representative experiment is shown.

Finally, we examined the effect of EGCG on HCV dsRNAs-induced ISGs expression in JFH-1-infected Huh7 cells. HCV dsRNAs also induced ISG15 and MxA at both mRNA (Fig. 7C–F, Fig. S4A,B) and protein (Fig. 6G, Fig. S4C) levels in JFH-1-infected Huh7 cells. Although EGCG alone had little effect on the expression of ISG15 (Fig. 7A) and MxA (Fig. 7B), EGCG treatment of HCV-infected cells significantly enhanced the HCV dsRNAs-induced ISG15 and MxA expression at both mRNA and protein levels in a dose-dependent manner (Fig. 7C–G and Fig. S4).

### Roles of TLR3 and RIG-I in HCV dsRNA-induced antiviral factors expression

**Figure 8:**
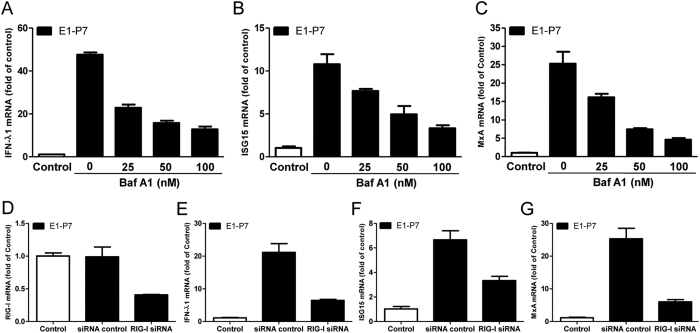
Roles of TLR3 and RIG-I signaling in HCV dsRNA-induced antiviral factors expression. (**A–C**) Huh7 cells were pretreated with Bafilomycin A1 (25–100 nM) for 1 h prior to E1-P7 stimulation (1 μg/mL). After 24 h E1-P7 stimulation, total RNA extracted from cells was subjected to the real-time RT-PCR for mRNA levels of IFN-λ1 (**A**), MxA (**B**) and ISG15 (**C**). (**D–G**) Huh7 cells were transfected with siRNA against RIG-I (100 nM) or siRNA control (100  nM) for 24 h prior to E1-P7 stimulation (1 μg/mL). After 24 h E1-P7 stimulation, total RNA extracted from cells was subjected to the real-time RT-PCR for mRNA levels of RIG-I (**D**), IFN-λ1 (**E**), ISG15 (**F**) and MxA (**G**). The data are expressed as mRNA levels stimulated for relative (fold) to the control (without stimulation, which is defined as 1). The results are mean ± SD of three different experiments.

To further determine whether TLR3 signaling pathway is critical in HCV dsRNA-induced IFN-λ1 and ISGs expression, Bafilomycin A1 (Baf A1) was used to block TLR3 signaling. As shown in Fig. 8A–C, HCV dsRNA (E1-P7) induced IFN-λ, ISG15 and MxA was compromised by Baf A1 treatment in a dose-dependent manner. In addition, siRNA against RIG-I was used to knockdown RIG-I expression in Huh7 cells. As shown in Fig. 8D, the knockdown efficiency of RIG-I mRNA expression by RIG-I siRNA was approximately 65% after 24 h transfection, and HCV dsRNA (E1-P7)-induced expression of IFN-λ (Fig. 8E), ISG15 (Fig. 8F) and MxA (Fig. 8G) was partially blocked in the cells transfected with RIG-I siRNA.

## Discussion

In the present study, we demonstrated that EGCG had the ability to enhance HCV dsRNAs-induced innate immune responses in hepatocytes. Although HCV dsRNAs could induce IFN-λ1 and ISGs expression in HCV-infected Huh7 cells, the induction levels was limited to inhibit viral replication. Our results revealed that EGCG significantly increased HCV dsRNAs-induced IFN-λ1, TLR3, RIG-I and several antiviral ISGs (ISG15 and MxA) expression in both JFH-1-infected and -uninfected Huh7 cells. More importantly, the combination of EGCG treatment and HCV dsRNA stimulation inhibited HCV replication in Huh7 cells. IFN-λ1 has been shown to have potent antiviral action on HCV replication and has been applied to clinical trials^20^,^21^. Although hepatocytes express a extremely low level of intracellular IFN-λ1, IFN-λ1 can be induced by TLR3 activation by its synthetical ligand or viral infections^22^,^23^. Our recent study^23^ showed that poly I:C stimulation induces the expression of IFN-λ1 in Huh7 cells. A recent study^22^ showed that HCV infection of primary liver cells stimulates expression of IFN-λ1, but not IFN-α or IFN-β. Furthermore, this production of IFN-λ1 was sufficient to inhibit HCV infection of primary hepatocytes^22^. Our data showed that EGCG enhanced HCV dsRNA-induced IFN-λ1 expression in JFH-1-infected Huh7 cells, which provides a sound mechanism for the combination of EGCG and HCV dsRNA-mediated HCV inhibition in Huh7 cells.

The interactions between HCV and the innate immunity in the liver play an important role in the immunopathogenesis of HCV disease. TLRs and RIG-I-like receptors (RLRs) are major cellular receptors that recognize pathogen-associated molecular patterns (PAMPs) during viral infections^24^. Several TLR family members participate in recognition of viral nucleic acids^25^. Among these TLRs members, TLR3 has a crucial role in virus-mediated innate immune responses^25^, as it recognizes viral dsRNA that either constitutes the genome of one class of viruses or is generated during the life cycle of viruses, including HCV dsRNAs^26^,^27^. RIG-I plays important role in HCV genome recognition, resulting in the activation of the type I IFN-dependent antiviral innate immune response to HCV infection^28^. It was reported that HCV dsRNA is readily detectable in Huh 7 cells that contain replicating HCV JFH-1 genome^29^. Here, we used *in vitro* synthesized HCV dsRNAs to stimulate Huh7 cells. We demonstrated that HCV dsRNAs induced TLR3, RIG-I and IFN-λ1 expression in Huh7 cells. However, the induction expression of IFN-λ1 by HCV dsRNAs was much lower than poly I:C in both mRNA and protein levels. Furthermore, we observed that HCV dsRNAs also induced a slightly expression of ISG15 and MxA in Huh7 cells. The important roles of TLR3 and RIG-I in recognition of HCV dsRNA and activation of antiviral factors expression were evidenced by the observation that Bafilomycin A1 and siRNA against RIG-I could block the action of HCV dsRNA intermediates.

The majority of HCV-infected subjects develop chronic infection, suggesting that HCV has evolved escape strategies for both innate and adaptive immunity of the target and immune cells in the liver^4^. Studies of HCV-host interactions have revealed that HCV NS3/4A protease is able to impair TLR3 by cleaving the Toll-IL-1 receptor domain-containing adaptor inducing IFN-β (TRIF) adaptor protein and block RIG-I signaling by cleaving the mitochondrial antiviral signaling protein (MAVS) off the mitochondria to inhibit IFN-β expression^5^,^6^,^7^. It is also shown that HCV infection can inhibit intracellular IFN-α and IFN-λ1 expression in hepatoma cells^23^,^30^. In addition, HCV infection could impair IRF-7 translocation and IFN-α synthesis in immortalized human hepatocytes^31^. Thus, it is important to recover the immune responses which have been compromised by exist viruses during viral transmission and replication. In this study, we demonstrated that EGCG, the major polyphenol component of green tea, can enhance *in vitro* synthesized HCV dsRNAs-induced innate immune responses in both HCV JFH-1-infected and -uninfected Huh7 cells. When we treated cells with EGCG before HCV dsRNAs stimulation, the HCV dsRNAs-induced IFN-λ1, RIG-I, TLR3 and several antiviral ISGs (ISG15, MxA) expressions were significantly enhanced, indicating that EGCG might be used as an agent to enhance the innate immune responses during viral infections.

The combination of PEG-IFN and ribavirin therapy was the standard of care for patients with chronic HCV infection with all genotypes for years^32^. However, this combination therapy has limited efficacy, depending on the different HCV genotypes. For instance, the sustained virologic response (SVR) reached by PEG-IFN and ribavirin combination therapy is higher among patients infected with genotype 2 and 3 than other genotypes^32^. Recently, new direct-acting antivirals (DAAs) were identified^33^. HCV NS3/4A protease inhibitors, boceprevir and telaprevir have been approved to supplement the current standard therapy, leading a triple therapy for HCV genotype 1 infection^33^,^34^. The management of chronic HCV infection changed dramatically with the approval of second-generation DDAs in 2013, which led the way for all-oral, IFN-free regimens with high rates of SVR, short duration of treatment and low side effect^35^. Despite the high efficacy of oral DAAs treatment, several challenges remain to be overcome in the future. Currently, high costs limit access to DAAs in most countries of the world. DAAs may have limitations in certain clinical scenarios such as advanced or decompensated liver disease and their use in children and pregnant women^36^. In the absence of a HCV vaccine, reinfection remains possible in patient populations with continued exposure to HCV after successful curative therapy^37^. In addition, DAAs lead to a high risk for selection of drug-resistant viral strains^38^. Thus, drugs that can enhance host immune responses which have been compromised by HCV would likely improve the SVR and eradicate the virus, such as EGCG.

In summary, EGCG not only potently inhibits HCV entry^18^,^19^, but also enhances HCV dsRNAs-induced antiviral innate immune responses. The clinical studies have shown that EGCG is safe and well tolerated in healthy human volunteers^39^. In the future, it would be interesting to investigate the anti-HCV property of EGCG in combination with current antiviral drugs for the treatment of patients chronically infected with HCV.

## Materials and Methods

### (**−**)-Epigallocatechin-3-gallate

EGCG (≥95%) was purchased from SIGMA-ALORICH St. Louis, MO, USA (CAS#: 989-51-5; Cat# E4143). EGCG stock solution was prepared in sterile double distilled water at 20 mM.

### Cells, plasmids, and virus

A hepatoma cell line (Huh7) provided by Dr. Charles Rice (The Rockefeller University, New York, NY, USA) was maintained in DMEM with 10% FBS, penicillin (100 U/mL), and streptomycin (100 μg/mL). The HCV pBSII-core, pBSII-E1-p7, pBSII-HCV-NS-3′NTR, pCMV-NS5A plasmids have been described^27^. HCV ssRNAs were synthesized from linearized vectors, using T7 and T3 MegaScript kits (Ambion, Inc., Austin, TX), as described previously^27^. To form dsRNA duplexes, equal molar amounts of + ssRNAs and -ssRNAs were mixed in a microcentrifuge tube and incubated at 100 °C for 5 minutes, followed by a slow cool-down to room temperature. The generation of infectious HCV JFH-1 and infection of Huh7 cells (MOI of 0.01) were carried out as previously described^40^. HCV JFH-1 infection of Huh7 was analyzed by the real time RT-PCR for HCV RNA.

### Reagents

LyoVec transfection reagent and poly I:C (HMW) were purchased from InvivoGen (San Diego, CA). ELISA kit for IFN-λ1 was purchased from eBioscience Inc. (San Diego, CA). Rabbit antibodies against RIG-I and ISG15 were purchased from Cell Signaling Technology, Inc. (Danvers, MA). Rabbit antibody against TLR3 was purchased from Novus Biologicals (Littleton, CO). Rabbit antibodies against MxA and Actin were purchased from SIGMA-ALORICH (St. Louis, MO). Bafilomycin A1 was purchased from EMD Chemical, Inc (Gibbstown, NJ). siRNA against RIG-I and negative control siRNA were purchased from Qiagen (Cambridge, MA).

### EGCG treatment and HCV dsRNAs stimulation

Huh7 cells were seeded at a density of 10^5^ each well in a 24 well-plate. After cultured for 24 h, the cells were treated with EGCG (1–10 μM) for 1 h prior to HCV dsRNAs (1 μg/mL) stimulation by LyoVec transfection reagent. Cells were collected for total RNA extraction after 24 h stimulation, and supernatant (SN) was collected for ELISA assay after 48 h stimulation. Cell lysates were collected for Western blot after 48 h stimulation. For the anti-HCV experiment, the JFH-1-infected Huh7 cells (72 h postinfection) were treated with EGCG (1–10 μM) for 1 h prior to HCV dsRNAs (1 μg/mL) stimulation. Culture SN and cell lysates were collected for total RNA extraction and protein purification after 48 h stimulation. As a negative control of the transfection experiment, cells were incubated with the LyoVec transfection reagent only.

### RNA extraction and real time RT-PCR

Total RNA from cultured cells or SN was extracted with Tri-Reagent (Molecular Research Center, Cincinnati, OH) as previously described^41^. Total RNA (1 μg) was subjected to RT using the RT system (Promega, Madison, WI) with random primers for 1 h at 42 °C. The reaction was terminated by incubating the reaction mixture at 99 °C for 5 min, and the mixture was kept at 4 °C. The resulting cDNA was used as a template for real time PCR quantification. Real time PCR was performed with 1/10 of the cDNA with the iQ SYBR Green Supermix (Bio-Rad Laboratories, Hercules, CA) as previously described^41^. The amplified products were visualized and analyzed using the software MyiQ provided with the thermocycler (iCycler iQ real time PCR detection system; Bio-Rad Laboratories). The oligonucleotide primers were synthesized by Integrated DNA Technologies, Inc. (Coralville, IA), and sequences were shown in Table 1. The cDNA was amplified by PCR and the products were measured using SYBR green I (Bio-Rad Laboratories, Inc., Hercules, CA). The data were normalized to glyceraldehyde-3-phosphate dehydrogenase (GAPDH) and presented as the change in induction relative to that of untreated control cells.

### Enzyme-linked immunosorbent assay

IFN-λ1 gene expression analyzed by the real time RT-PCR was evaluated by ELISA for protein expression. SN collected from EGCG and/or HCV dsRNAs-treated Huh7 cell cultures was directly tested for IFN-λ1 protein levels by ELISA, which was performed according to the manufacturer’s instructions.

### Western blot analysis

The expression of the TLR3, RIG-I, ISG15, MxA, and Actin protein was evaluated by immunoblot analysis. Following incubation with specific antibodies and extensive washing in PBS containing 0.05% Tween-20, membranes were incubated with horseradish peroxidase-conjugated goat anti-rabbit IgG (Pierce, Chester, UK) for 1 h at room temperature. Membranes were extensively washed in PBS containing 0.05% Tween-20, and immunoblots were visualized by Fujifilm Las-1000 Luminescent Image Analyzer (Fujifilm,Tokyo, Japan).

## Additional Information

**How to cite this article**: Wang, Y. *et al*. (−)-Epigallocatechin-3-Gallate Enhances Hepatitis C Virus Double-Stranded RNA Intermediates-Triggered Innate Immune Responses in Hepatocytes. *Sci. Rep.* **6**, 21595; doi: 10.1038/srep21595 (2016).

## Supplementary Material

Supplementary Information

## Figures and Tables

**Table 1:** Primers for real-time RT-PCR.

Primer	Orientation	Sequences (5′–3′)
GAPDH	Forward	GGTGGTCTCCTCTGACTTCAACA
	Reverse	GTTGCTGTAGCCAAATTCGTTGT
IFN-λ1	Forward	CTTCCAAGCCCACCCCAACT
	Reverse	GGCCTCCAGGACCTTCAGC
TLR3	Forward	AGCCACCTGAAGTTGACTCAGG
	Reverse	CAGTCAAATTCGTGCAGAAGGC
RIG-I	Forward	CTTGGCATGTTACACAGCTGAC
	Reverse	GCTTGGGATGTGGTCTACTCA
ISG15	Forward	GGCTGGGAGCTGACGGTGAAG
	Reverse	GCTCCGCCCGCCAGGCTCTGT
MxA	Forward	GCCGGCTGTGGATATGCTA
	Reverse	TTTATCGAAACATCTGTGAAAGCAA
HCV	Forward	RAYCACTCCCCTGTGAGGAAC
	Reverse	TGRTGCACGGTCTACGAGACCTC
