# Supplementary material for: (−)-Epigallocatechin-3-Gallate Enhances Hepatitis C Virus Double-Stranded RNA Intermediates-Triggered Innate Immune Responses in Hepatocytes
Source: Sci Rep. 2016 Feb 16;6:21595. doi: 10.1038/srep21595 (PMC4754899; doi:10.1038/srep21595)
Supplement: Supplementary Information [file srep21595-s1.doc]

(−)**-Epigallocatechin-3-Gallate Enhances Hepatitis C Virus Double-Stranded RNA Intermediates-Triggered Innate Immune Responses in Hepatocytes**

Yizhong Wang^1*^, Jieliang Li^2^, Xu Wang^2^ , Juliet C. Peña^2^, Kui Li^3^, Ting Zhang^1*^ and Wenzhe Ho^2*^

^1^Department of Infectious Diseases, Shanghai Children's Hospital, Shanghai Jiao Tong University, Shanghai 200040, P.R. China.

^2^Department of Pathology and Laboratory Medicine, Temple University School of Medicine, Philadelphia, PA 19140, USA.

^3^Deparment of Microbiology, Immunology, and Biochemistry, University of Tennessee Health Science Center, Memphis, TN 38163, USA.

**Supplementary information**

**Figure S1. EGCG enhances HCV dsRNAs-induced IFN-λ1 expression in Huh7 cells.** Huh7 cells were treated with EGCG as indicated for 1 h prior to HCV dsRNAs (Core, NS5A) stimulation. Total RNA extracted from cells after 24 h stimulation was subjected to the real time RT-PCR for the mRNA levels of IFN-λ1 and GAPDH. The data are expressed IFN-λ1 mRNA (A, B) levels relative (fold) to the control (vehicle only, which defined as 1). After 48 h stimulation, supernatant (SN) was collected from the cell cultures for ELISA to measure the protein level of IFN-λ1 (C). The results shown are mean ± SD of triplicate, representative of three independent experiments.

**Figure S2. EGCG increases HCV dsRNAs-induced TLR3 and RIG-I expression.** Huh7 cells were treated with EGCG as indicated for 1 h prior to HCV dsRNAs (Core, NS5A) stimulation. Total RNA extracted from cells after 24 h stimulation was subjected to the real time RT-PCR for the mRNA levels of TLR3 and RIG-I and GAPDH. The data are expressed TLR3 (A, B) and RIG-I mRNA (C, D) levels relative (fold) to the control (vehicle only, which defined as 1). The results shown are mean ± SD of triplicate, representative of three experiments. After 48 h stimulation, cell lysates were collected from the cell cultures for Western blot to measure the protein level of TLR3 (E) and RIG-I (F). Three independent experiments were performed and one representative experiment is shown.

**Figure S3. EGCG enhances HCV dsRNAs-induced ISGs expression in Huh7 cells.** Huh7 cells were treated with EGCG as indicated for 1 h prior to HCV dsRNAs (Core, NS5A) stimulation. Total RNA extracted from cells after 24 h stimulation was subjected to the real time RT-PCR for the mRNA levels of ISG15 and MxA and GAPDH. The data are expressed ISG15 (A, B) and MxA mRNA (C, D) levels relative (fold) to the control (vehicle only, which defined as 1). The results shown are mean ± SD of triplicate, representative of three experiments. After 48 h stimulation, cell lysates were collected from the cell cultures for Western blot to measure the protein level of ISG15 and MxA (E). Three independent experiments were performed and one representative experiment is shown.

**Figure S4. Effect of EGCG on HCV dsRNAs-induced ISGs expression in JFH-1-infected Huh7 cells.** JFH-1-infected Huh7 cells (72 h postinfection) were treated with EGCG as indicated for 1 h prior to HCV dsRNAs (Core, NS5A) stimulation. Total RNA extracted from cells after 24 h stimulation was subjected to the real time RT-PCR for the mRNA levels of ISG15 and MxA and GAPDH. The data are expressed ISG15 (A) and MxA mRNA (B) level relative (fold) to the control (vehicle only, which defined as 1). The results shown are mean ± SD of triplicate, representative of three experiments. After 48 h stimulation, cell lysates were collected from the cell cultures for Western blot to measure the protein levels of ISG15 and MxA (C). Three independent experiments were performed and one representative experiment is shown.
